# Mitochondrial remodeling after conventional and ultra-high dose rate irradiation in head and neck carcinoma cells in vitro

**DOI:** 10.1016/j.ctro.2026.101252

**Published:** 2026-08-06

**Authors:** Liliana L. Silva, Tianshi Liu, Filip Hörberger, Imen Chamkha, Nora Li, Crister Ceberg, Eskil Elmér, Gabriel Adrian, Johannes K. Ehinger

**Affiliations:** a Department of Medical Radiation Physics, Department of Clinical Sciences Lund, Lund University, Lund, Sweden; b Mitochondrial Medicine, Department of Clinical Sciences Lund, Lund University, Lund, Sweden; c Division of Oncology and Pathology, Department of Clinical Sciences Lund, Skåne University Hospital, Lund, Sweden; d Otorhinolaryngology, Head and Neck Surgery, Department of Clinical Sciences Lund, Lund University, Lund, Sweden; e Otorhinolaryngology, Skåne University Hospital, Lund, Sweden

**Keywords:** FLASH radiotherapy, ultra-high dose rate, head and neck squamous cell carcinoma, mitochondria, high-resolution respirometry, radioresistance

## Abstract

**Background and purpose:**

Ultra-high dose rate (UHDR) irradiation is being investigated as a strategy to widen the therapeutic window of radiotherapy, but the biological mechanisms that distinguish UHDR from conventional dose rate (CONV) irradiation remain unresolved. Because mitochondria integrate bioenergetics, redox control and stress signaling after irradiation, they represent a plausible site at which dose-rate effects could emerge. This study evaluated mitochondrial function after CONV and UHDR irradiation in a head and neck squamous cell carcinoma in vitro model.

**Materials and methods:**

LU-HNSCC4 (HN4, an oral cavity HNSCC cell line) cells and VH10 fibroblasts were treated with 0, 2, 6, 10 or 20 Gy delivered at CONV or UHDR using electron irradiation from a modified linear accelerator. High-resolution respirometry was performed immediately after irradiation and 48 h later. In the 0 and 20 Gy groups, flow cytometry was used to assess cellular reactive oxygen species (ROS), mitochondrial ROS (mtROS), mitochondrial membrane potential (MMP) and Magnesium Green fluorescence as an indirect ATP-related readout. RNA sequencing was performed in HN4 cells 48 h after 20 Gy.

**Results:**

Irradiation induced clear dose- and time-dependent increases in oxygen consumption in HN4 cells, affecting routine respiration, oxidative phosphorylation capacity and maximal electron transfer capacity. The effect was strongest at higher doses and at 48 h and involved both complex I- and complex II-linked pathways. No statistically significant differences between dose-rate modalities were detected for the principal respiratory endpoints. Flow-cytometric analyses at 20 Gy showed stress responses in both irradiation arms, with some possible differences in temporal pattern; but without a clearly distinct overall phenotype attributable to dose rate.

**Conclusion:**

The data support a model in which both CONV and UHDR engage a broadly similar post-irradiation mitochondrial adaptive program in HN4 cells. The findings are relevant to the study of radiation adaptation and support further testing of mitochondria-directed radiosensitization strategies in head and neck cancer.

## Highlights

• Irradiation induced marked dose- and time-dependent mitochondrial remodeling in HN4 head and neck cancer cells.
• CONV and UHDR produced broadly similar overall mitochondrial phenotypes, with limited evidence for a distinct UHDR-specific effect.
• CONV was associated with an earlier oxidative/metabolic response, whereas UHDR was characterized mainly by delayed mtROS elevation.
• RNA sequencing showed substantial overlap between dose rates, including downregulation of mitochondrial gene sets.

## 1 Introduction

Radiotherapy remains a cornerstone of cancer treatment, but its efficacy is constrained by normal-tissues toxicities and by intrinsic or acquired tumor radioresistance [1], [2], [3]. In head and neck squamous cell carcinoma (HNSCC), these challenges are clinically important, with loco-regional control relying on high radiotherapy doses, often associated with substantial treatment-related morbidity [4].

Ultra-high dose rate (UHDR) irradiation, often referred to as FLASH, has attracted strong interest because preclinical studies suggest that ultra-high dose-rate delivery can reduce normal-tissue toxicity while preserving tumor control [5], [6]. At the same time, the mechanistic basis of the FLASH effect remains unsettled. Oxygen depletion, altered radical chemistry, immune effects and mitochondrial responses have all been proposed, and available data indicate that any dose-rate effect is likely to be highly context dependent [5], [6], [7], [8].

Mitochondria are particularly relevant in this setting. Ionizing radiation perturbs mitochondrial redox balance, respiratory chain function, membrane potential and cell-death signaling, and these responses can shape both radiosensitivity and post-irradiation adaptation [9], [10], [11]. Mitochondria are a plausible contributor to the FLASH/UHDR effect because they integrate several processes already implicated in current mechanistic models, including oxygen handling, reactive oxygen species (ROS) production, redox buffering, ATP homeostasis, and stress signaling. If UHDR irradiation alters the early oxidative chemistry of radiation exposure, this may secondarily modify mitochondrial injury and the downstream metabolic response. In that case mitochondria could represent an important biological node through which differences in oxygen tension and radical chemistry are translated into differences in cellular survival and tissue toxicity. In cancer, mitochondrial metabolism is also linked to metabolic plasticity, oxidative stress buffering and resistance to treatment [10], [11], [12], [13].

These considerations led us to test whether UHDR and CONV irradiation produce distinct mitochondrial phenotypes in HN4 head and neck cancer cells and in VH10 fibroblast as non-malignant control cells.

## 2 Materials and methods

### 2.1 Cell culturing and irradiation

HN4 (LU-HNSCC4) head and neck squamous cell carcinoma cells [14] and VH10 fibroblasts (RRID:CVCL_RW72) were cultured as previously described [15] in 37 °C in an incubator with a 5% CO_2_ in high-glucose Dulbecco's modified Eagle medium (DMEM; Sigma-Aldrich, Schnelldorf, Germany) supplemented with 10% fetal bovine serum (FBS) and 1% of penicillin-streptomycin (Sigma-Aldrich, Schnelldorf, Germany). Irradiation was performed with a modified Elekta Precise linear accelerator enabling 10 MeV electron UHDR delivery [16], [17]. UHDR was delivered in 3.5 microsecond pulses at 200 Hz with a dose per pulse of 2.0 Gy, corresponding to an average dose rate of approximately 400 Gy/s; CONV irradiation was delivered at approximately 0.1 Gy/s. Total doses were 0, 2, 6, 10 and 20 Gy. Gafchromic EBT3 film were used to verify dose as previously described [16], [17].

### 2.2 Mitochondrial respirometry

Mitochondrial respiration was assessed by high-resolution respirometry (O2k, Oroboros Instruments) immediately after irradiation (within 1 h after irradiation) and 48 h later using a substrate-uncoupler-inhibitor titration protocol adapted from prior work [18], [19]. The principal endpoints used in the main analysis and displayed in figures were routine respiration (oxygen consumption under idle conditions without additions), maximum oxidative phosphorylation (OXPHOS) capacity (ADP-stimulated OXPHOS with substrates for both mitochondrial complex I and complex II present) and maximal electron transfer (ET) capacity (maximum FCCP induced uncoupled oxygen consumption with substrates for both mitochondrial complex I and complex II present) . Supplementary analyses included complex I-linked OXPHOS, complex II-linked ET, LEAK respiration, oligomycin sensitive ATP-linked respiration and the OXPHOS/ET ratio.

### 2.3 Flow cytometry

For the 0 and 20 Gy groups, flow cytometry was used to assess cellular ROS (DHE), mitochondrial ROS (mtROS; MitoSOX), MMP (TMRM) and Magnesium Green fluorescence as an indirect ATP-related readout in HN4 cells, using a BD FACSymphony A1. The 20 Gy condition was selected for the flow cytometry experiments (and the subsequent RNA sequencing) because it produced the clearest irradiation-associated changes in the respirometry dataset. All incubations were performed in pre-warmed mitochondrial respiration medium (MiR05) at a cell-density density of 1 million cells/mL. After addition of each probe, cells were incubated in the dark for 20 min. After incubation, cells were washed and resuspended in pre-warmed MiR05 solution. Flow-cytometric analyses were performed at time points 0 h (experiments performed within 1 h after irradiation) and at 48 h, with *n* = 6 independent experiments per condition.

For mtROS, 5 μM MitoSOX Red (Thermo Fisher Scientific via Life Technologies Europe BV, Stockholm, Sweden) was used, at an excitation/emission (ex/em) wavelength of 510/586 nm. For Dihydroethidium (DHE) (Thermo Fisher Scientific via Life Technologies Europe BV, Stockholm, Sweden), a 10 μM concetration was used at an ex/em wavelength of 561/610 nm. For the indirect measure of cellular ATP 1 μM Magnesium Green AM Cell Permeant (Catalog#. M3735, Thermo Fisher Scientific via Life Technologies Europe BV, Stockholm, Sweden) (MgGr) was used. MgGr fluoresces upon binding to free Mg2+, which exhibits a higher affinity for ATP compared to ADP. Ex/em wavelengths of 405/605 nm was used. To assess ΔΨm 20 nM TMRM (Tetramethylrhodamine, Methyl Ester, Perchlorate (TMRM) (Catalog#: T668), Thermo Fisher Scientific via Life Technologies Europe BV, Stockholm, Sweden) was added, using ex/em 405/586. The data analysis was performed using FlowJo v10 Software (BD Life Sciences) [20].

### 2.4 RNA sequencing

RNA sequencing was performed by Active Motif Europe (Waterloo, Belgium) (https://www.activemotif.com) at time point 48 h in HN4 cells exposed to CONV 20Gy or UHDR 20Gy. 2 million cells per condition were stored at −80 °C and subsequently RNA was extracted using the MagMAX™ mirVana™ Total RNA Isolation Kit (cat#: A27828) on the KingFisher Apex instrument (ThermoFisher 5,400,930). For each sample, 2 μg of total RNA was then used in Illumina's TruSeq Stranded mRNA Library kit (Cat# 20020594).

### 2.5 Statistical analysis

The statistical analyses on respirometry and flow cytometry data were performed using GraphPad Prism 10, using a 2 way-ANOVA with Bonferroni's multiple comparisons test within each analysis, followed by a False Discovery Rate (FDR) analysis with the two-stage step-up method of Benjamini, Kriefger and Yekutieli, involving results from all the tests performed in the study to identify the most significative discovery. The data are presented as mean values with standard error of the mean (SEM); *n* = 6 per condition and *p < 0.05, **p < 0.01, ***p < 0.001 and ****p < 0.0001. The graphics were produced using GraphPad Prism 10 and the images using Inskape Draw Freely v 1.3.

The differential gene expression analysis was performed to identify statistically significant differential genes using DESeq2. The Gene enrichment analysis (GSEA) was performed using clusterProfiler and the gene ontology (GO) analysis was performed for the selected gene sets to identify enriched functional categories.

## 3 Results

### 3.1 Irradiation increased mitochondrial respiration in HN4 cells but not in VH10 fibroblasts

**Fig. 1:**
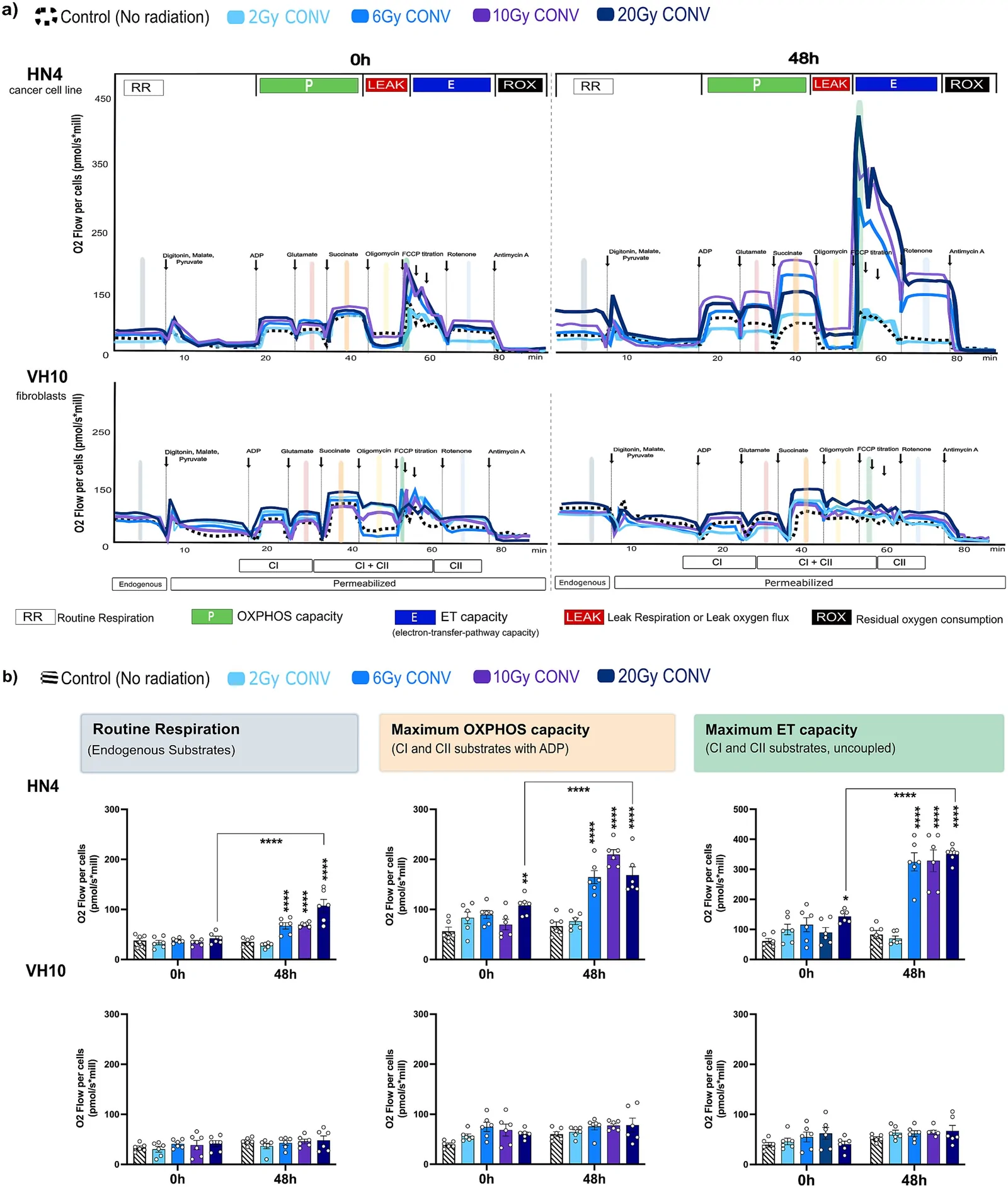
Oxygen consumption in HN4 cancer cells and VH10 fibroblasts cells with conventional (CONV) irradiation depending on total Gy. **a)** Representative respirometry charts of HN4 cells and VH10 after irradiation with CONV 0/ 2/6/10 and 20Gy at 0 h (immediately after irradiation) and 48 h after irradiation. **b)** Routine respiration (with endogenous substrates), OXPHOS and ET, both Complex I- and Complex II-linked substrates. All values were corrected for residual oxygen consumption ROX. Data are presented as mean ± standard error of the mean (SEM); *n* = 6 per condition and *p < 0.05, **p < 0.01, ***p < 0.001 and **** *p* < 0.0001 from FDR analysis (most significative discovery).

**Fig. 2:**
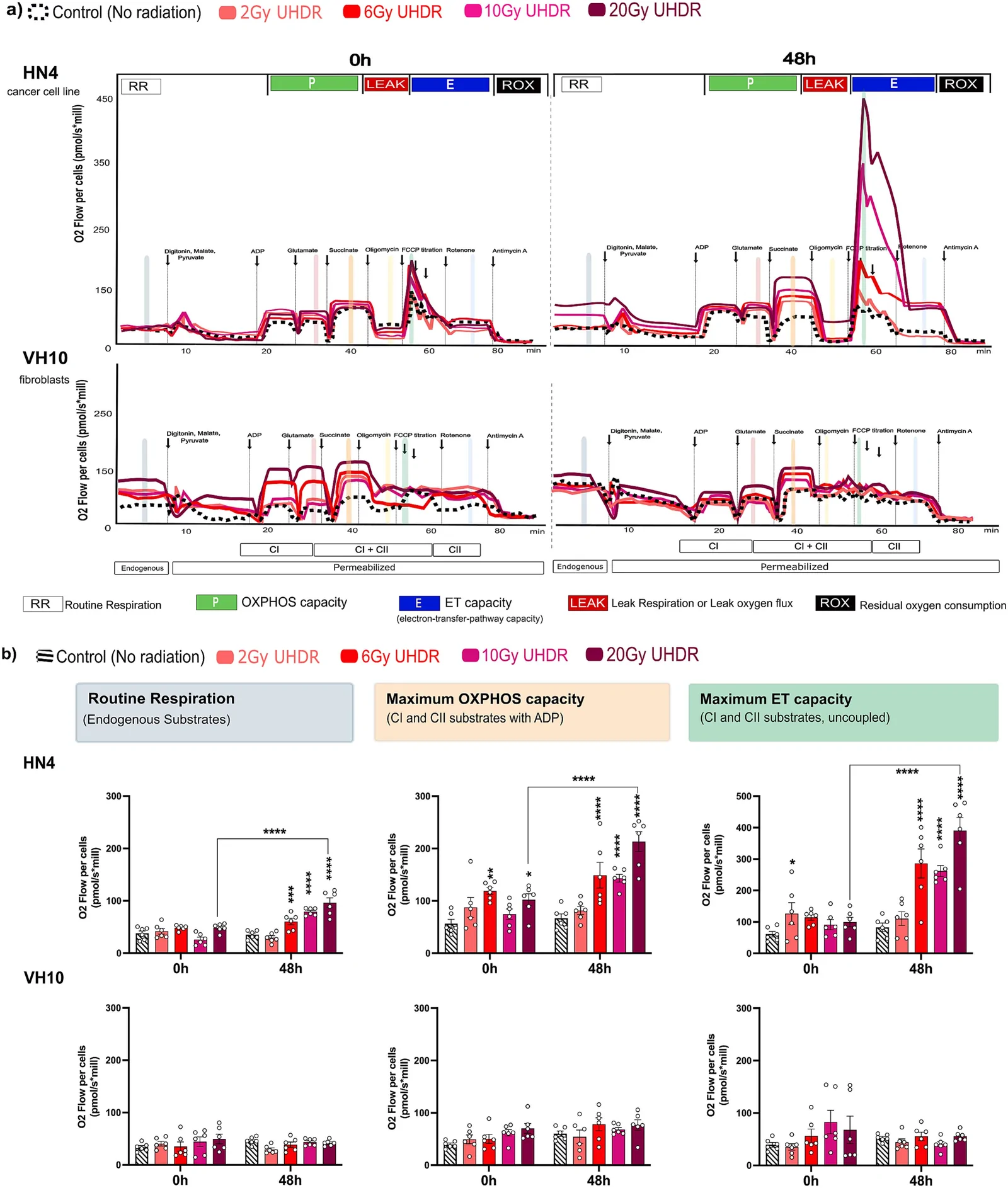
Oxygen consumption in HN4 cancer cells and VH10 fibroblasts cells with ultra-high dose rate (UHDR) irradiation depending on total Gy. **a)** Representative respirometry charts of HN4 cells and VH10 after irradiation with UHDR 0/ 2/6/10 and 20Gy at 0 h (immediately after irradiation) and 48 h after irradiation. **b)** Routine respiration (with endogenous substrates), OXPHOS and ET, both Complex I- and Complex II-linked substrates. All values were corrected for residual oxygen consumption ROX. Data are presented as mean ± standard error of the mean (SEM); *n* = 6 per condition and *p < 0.05, **p < 0.01, ***p < 0.001 and **** *p* < 0.0001 from FDR analysis (most significative discovery).

In HN4 cells, both CONV (Fig. 1) and UHDR (Fig. 2) produced dose- and time-dependent increases in routine respiration, OXPHOS capacity and ET capacity. The effect was modest immediately after irradiation but became pronounced at 48 h, especially after 6–20 Gy. Supplementary analyses (Fig. S1; S2) showed that the increase was not restricted to a single respiratory state but extended to ATP-linked respiration and to both complex I- and complex II-supported pathways. In contrast, VH10 fibroblasts displayed virtually no changes. This contrast between tumor and fibroblast cells indicates an irradiation-induced mitochondrial adaptation specifically in the malignant cells rather than a general response shared by both cell types.

### 3.2 UHDR and CONV produced broadly similar respiratory phenotypes

When the two dose-rate modalities were compared directly at each time point and dose, oxygen consumption in HN4 cells was often numerically higher after UHDR, but the principal respiratory endpoints did not differ significantly between UHDR and CONV. Thus, the data do not support a strong dose-rate-specific mitochondrial effect at the level of integrated respiration in this model. Instead, they show that irradiation itself, independent of dose rate, is the major determinant of the mitochondrial phenotype observed here.

### 3.3 The OXPHOS/ET ratio decreased after high-dose irradiation

In the 20 Gy groups, the ratio between OXPHOS capacity and maximal uncoupled ET capacity decreased from 0 h to 48 h after both CONV and UHDR (Fig. S3). This pattern suggests that, by 48 h, the phosphorylation system contributes relatively more to respiratory limitation than upstream electron transfer. This observation adds mechanistic nuance to the respirometry data and is consistent with a state of persistent mitochondrial upregulation in which ATP synthesis capacity becomes relatively constrained.

### 3.4 Flow cytometry suggested different timing, but not a different overall direction, of the stress response

**Fig. 3:**
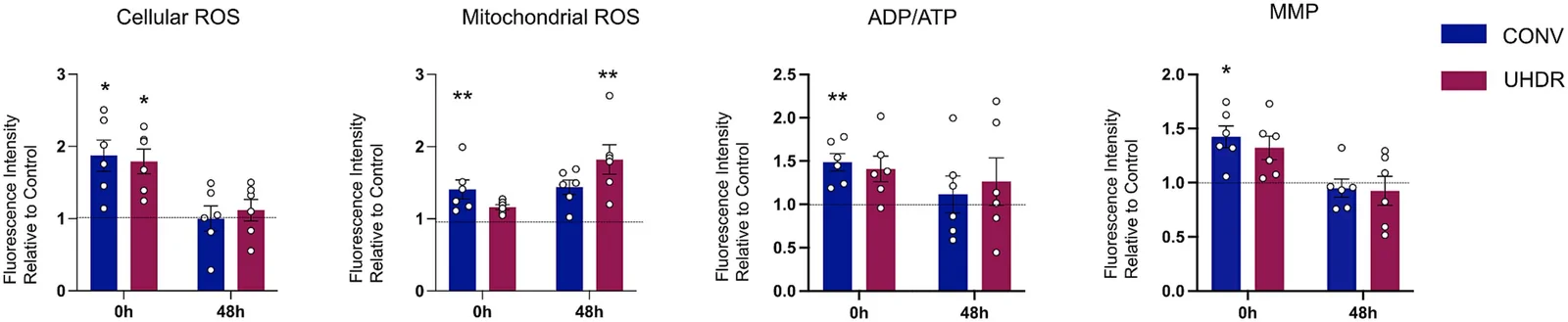
Reactive oxygen species, ATP-levels and Mitochondrial Membrane Potential. **a)** Cellular ROS using DHE (excitation/emission wavelength 561/586 nm). **b)** Mitochondrial ROS (mtROS) using MitoSOX (Ex/Em 561/610 nm). **c)** Indirect quantification of ADP/ATP using MgGr (Ex/Em 405/605 nm). **d)** Mitochondrial Membrane Potential (MMP) using TMRM (Ex/Em 561/586 nm). Data expressed as the ratio of fluorescence intensity in the experimental groups relative to control. All assays done in HN4 (1,000,000 cells/mL cells) 0 h and 48 h after irradiation with CONV 20Gy and UHDR 20Gy using a BD FACSymphony A1 flow cytometer. Control samples were not irradiated, *n* = 6 per condition. The bars represent mean values ± standard error of the mean (SEM); n = 6 per condition and *p < 0.05, **p < 0.01, ***p < 0.001 and **** *p* < 0.0001 compared to control.

At 20 Gy, both dose rates triggered oxidative and metabolic perturbation in HN4 cells (Fig. 3). Some markers deviated from control earlier after CONV and later after UHDR. Cellular ROS increased immediately after both CONV and UHDR and returned toward baseline by 48 h. CONV induced an early increase in mtROS, a reduction in ATP-related signal and an increase in MMP immediately after irradiation, with normalization by 48 h. UHDR showed less evidence of an immediate response in these assays, but mtROS increased at the later time point. However, these patterns should be interpreted as descriptive temporal differences within each irradiation arm rather than as evidence of differences between dose-rate modalities.

### 3.5 RNA sequencing indicated broad mitochondrial remodeling after irradiation

**Fig. 4:**
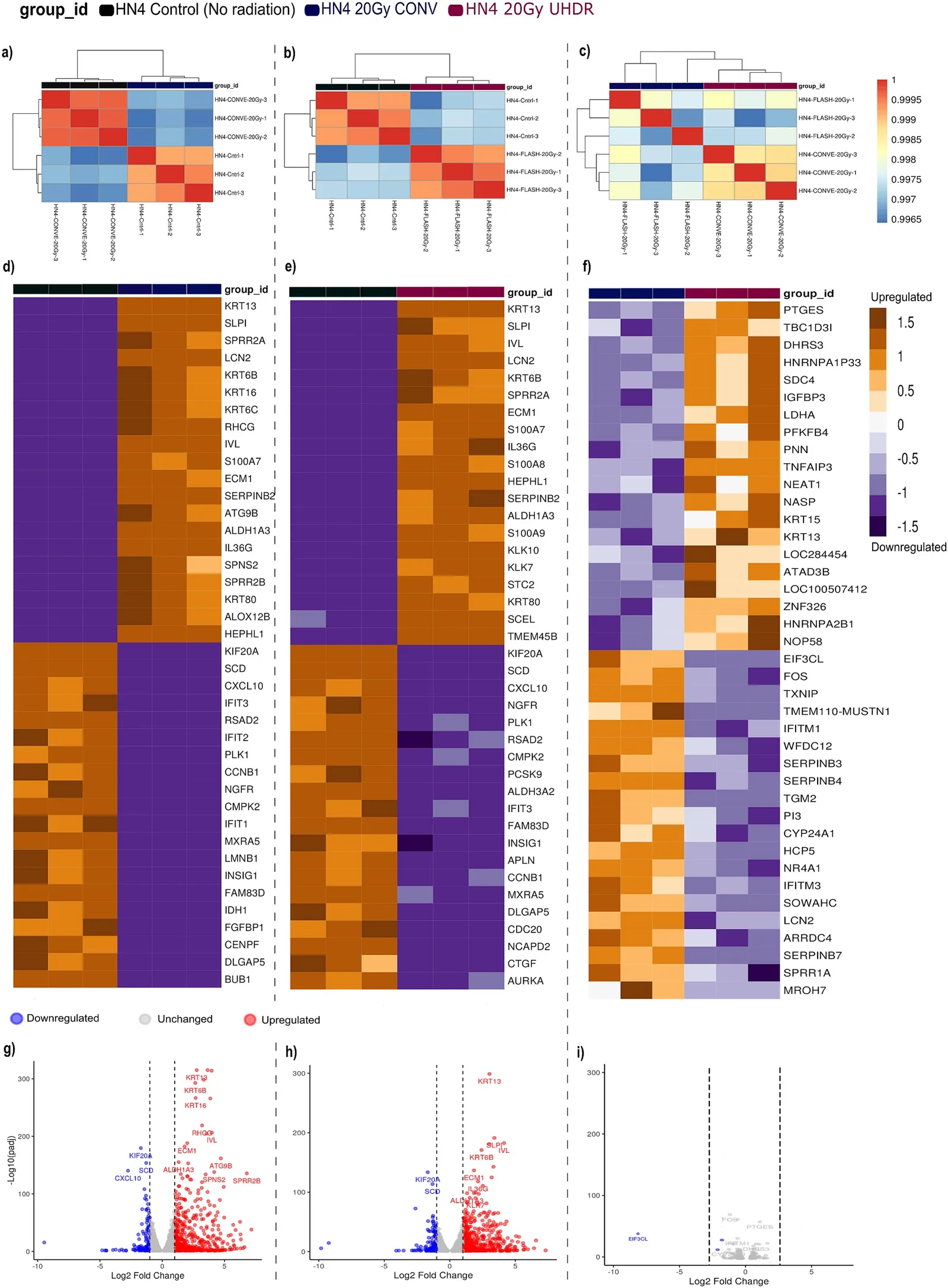
RNA-expression in HN4 cells 48 h after 20 Gy total dose of CONV or UHDR irradiation. Heatmap of Pearson Correlation, using the top 20 variable genes, between: **a)** non-irradiated samples (Control) and CONV 20Gy, **b)** Control and UHDR 20Gy, and **c)** CONV 20Gy and UHDR 20 Gy. Heatmap of the top 20 differentially expressed between: **d)** Control and CONV 20Gy, **e)** Control and UHDR 20Gy, and **f)** CONV 20Gy and UHDR 20 Gy. Volcano plot of the differentially expressed genes comparing **g)** Control and CONV 20Gy, **h)** Control and UHDR 20Gy, and **i)** CONV 20Gy and UHDR 20.

Transcriptomic analysis at 48 h revealed substantial overlap between CONV and UHDR, with downregulation of gene sets related to mitochondrial membranes, mitochondrial matrix, mitochondrial ribosome and oxidoreductase activity (Fig. 4, Fig. S4).

## 4 Discussion

This study demonstrates that irradiation induces a pronounced mitochondrial adaptive response in HN4 head and neck cancer cells, whereas VH10 fibroblasts remain comparatively stable. This response does not differ with different dose rates. By 48 h after irradiation, HN4 cells exhibited higher routine respiration, OXPHOS capacity and ET capacity across a range of doses, with additional support from pathway-specific analyses in the supplementary dataset. These findings fit well with, and complement, the broader literature linking mitochondrial function to radiation response, redox adaptation and treatment resistance [10], [11], [12], [13], [21], [22], [23].

Our results do not provide support for a dose-rate-specific mitochondrial mechanism under the tested conditions. UHDR biology is highly dependent on dose-rate metrics, beam structure, oxygenation, tissue context and endpoint selection [5], [6], [7], [8]. The present negative findings are still an important contribution to the mechanistic study of FLASH/UHDR irradiation. While HN4 cells showed pronounced irradiation-induced mitochondrial remodeling, we found no direct difference between CONV and UHDR in mitochondrial respiration, ATP-related signal, mitochondrial membrane potential, or ROS-related measurements. This argues against a simple model in which UHDR exerts its biological effects through an immediate and intrinsic alteration of mitochondrial function in this normoxic in-vitro system. Our data therefore suggest that if mitochondria contribute to FLASH biology, that contribution is likely to be indirect, conditional, or dependent on additional biological factors not captured in the present model.

A potential weakness of this study is that all experiments were performed under ambient oxygen. Previous work with HN4 and related models has shown that UHDR responses can vary across tumor types and oxygenation conditions [17], [24], and it cannot be excluded that the results could have looked different in hypoxic conditions, as is often the case in the tumor micro-environment. Accordingly, the absence of a distinct mitochondrial phenotype in the present setting should not be interpreted as evidence against FLASH biology in general. Rather, we have shown that the radiation response of mitochondria in an in vitro system under normoxic conditions do not differ between dose rates. The study was also limited to one HNSCC model and one fibroblast model. Accordingly, the observed contrast between HN4 and VH10 can't be interpreted as a general distinction between all malignant and non-malignant cells. The flow-cytometric and RNA-seq data add useful context but should also be interpreted cautiously. The flow-cytometric assays were restricted to 20 Gy, and the Magnesium Green signal is only an indirect ATP-related readout. Likewise, RNA sequencing at a single late time point cannot by itself explain the functional rise in respiration. Rather, the transcriptomic results should be viewed as evidence of broad mitochondrial and metabolic remodeling after irradiation. Repeated sampling and molecular analysis would be valuable in future work.

We recently showed that HNSCC cell lines differ markedly in oxidative and glycolytic reserve depending on human papilloma virus (HPV) status, and that HPV-negative HNSCC cells, including the LU-HNSCC4, rely more heavily on mitochondrial oxidative phosphorylation compared to HPV-positive cancer cells [15]. Although that question was not addressed here, such metabolic heterogeneity may be relevant to how different HNSCC models respond to radiation-induced bioenergetic stress. HPV-positive oropharyngeal cancers are more radiosensitive than HPV negative, and it is plausible that the metabolic profile influences how tumor cells tolerate radiation-induced bioenergetic and redox stress [4], [15].

The study has translational implications. A principal significance of the present study lies in showing that irradiation itself drives a robust mitochondrial adaptive response in this HNSCC model. Because mitochondrial adaptation may contribute to post-irradiation survival, mitochondria-directed radiosensitization remains an attractive strategy in HNSCC [4], [10], [11], [12], [13], [21], [22], [23]. Inhibiting complex I is one such approach. Prior work has shown that metformin can inhibit complex I-dependent mitochondrial metabolism [22], and a recent systematic review supports continued interest in metformin as a radiosensitizer, although mechanisms are likely multifactorial [23]. This data set adds further relevance to future testing of mitochondrial inhibition as a mean to overcome radioresistance in HNSCC.

## 5 Conclusion

Irradiation with both conventional and ultra-high dose rates induced substantial mitochondrial remodeling in HN4 head and neck cancer cells, with limited changes in VH10 fibroblasts. The dominant pattern was a dose- and time-dependent increase in mitochondrial respiratory capacity accompanied by redox and transcriptomic adaptation. Under these normoxic in vitro conditions, UHDR did not produce a distinct mitochondrial respiration phenotype compared with CONV. The study therefore supports a careful conclusion: mitochondria are centrally involved in the adaptive response of irradiated HN4 cells, but this model does not support that mitochondrial function explains putative dose-rate-specific irradiation effects.

## Consent for publication

Not applicable.

## Ethics approval and consent to participate

Not applicable.

## Funding

This study was supported by The Royal Physiographic Society of Lund - Endowments for the Natural Sciences, Medicine and Technology (2023) (Application 44267), the Swedish Cancer Society (201298 Pj01H and 211929S01H), Mrs. Berta Kamprad Foundation (FBKS-2024-23 -(608)).

## Declaration of generative AI and AI-assisted technologies in the manuscript preparation process

During the preparation of this work, the authors used ChatGPT (OpenAI) to assist with language editing, restructuring of the manuscript text, clarification of the presentation of results, and improvement of contextual discussion of the findings in relation to the literature. The authors reviewed, edited, and verified all generated text and references and take full responsibility for the content of the published article.

## Declaration of competing interest

The authors declare that they have no known competing financial interests or personal relationships that could have appeared to influence the work reported in this paper.

## References

[1] De Ruysscher D., Niedermann G., Burnet N.G. (2019). Radiotherapy toxicity. Nat Rev Dis Primers.

[2] Citrin D.E. (2017). Recent developments in radiotherapy. N Engl J Med.

[3] Joiner M., van der Kogel A. (2024).

[4] Hutchinson M.K.N.D., Mierzwa M.L., D’Silva N.J. (2020). Radiation resistance in head and neck squamous cell carcinoma: dire need for an appropriate sensitizer. Oncogene.

[5] Adrian G., Konradsson E., Lempart M., Bäck S., Ceberg C., Petersson K. (2020). The FLASH effect depends on oxygen concentration. Br JRadiol.

[6] Petersson K., Adrian G., Butterworth K., McMahon S.J. (2020). A quantitative analysis of the role of oxygen tension in FLASH radiation therapy. Int J Radiat Oncol Biol Phys.

[7] Limoli C.L., Vozenin M.C. (2023). Reinventing radiobiology in the light of FLASH radiotherapy. Annu Rev Cancer Biol.

[8] Bogaerts E., Macaeva E., Isebaert S., Haustermans K. (2022). Potential molecular mechanisms behind the ultra-high dose rate FLASH effect. Int J Mol Sci.

[9] Riley P.A. (1994). Free radicals in biology: oxidative stress and the effects of ionizing radiation. Int J Radiat Biol.

[10] Zaffaroni M., Vincini M.G., Corrao G. (2022). Unraveling mitochondrial determinants of tumor response to radiation therapy. Int J Mol Sci.

[11] Ding Y., Jing W., Kang Z., Yang Z. (2025). Exploring the role and application of mitochondria in radiation therapy. Biochim Biophys Acta Mol basis Dis.

[12] Tang L., Wei F., Wu Y. (2018). Role of metabolism in cancer cell radioresistance and radiosensitization methods. J Exp Clin Cancer Res.

[13] Wallace D.C. (2012). Mitochondria and cancer. Nat Rev Cancer.

[14] Henriksson E., Baldetorp B., Borg A. (2006). p53 mutation and cyclin D1 amplification correlate with cisplatin sensitivity in xenografted human squamous cell carcinomas from head and neck. Acta Oncol.

[15] Li N., Chamkha I., Verma G. (2024). Human papillomavirus-associated head and neck squamous cell carcinoma cells rely on glycolysis and display reduced oxidative phosphorylation. Front Oncol.

[16] Lempart M., Blad B., Adrian G. (2019). Modifying a clinical linear accelerator for delivery of ultra-high dose rate irradiation. Radiother Oncol.

[17] Adrian G., Konradsson E., Beyer S., Wittrup A., Butterworth K.T., McMahon S.J. (2021). Cancer cells can exhibit a sparing FLASH effect at low doses under normoxic in vitro conditions. Front Oncol.

[18] Piel S., Chamkha I., Dehlin A.K. (2020). Cell-permeable succinate prodrugs rescue mitochondrial respiration in cellular models of acute acetaminophen overdose. PLoS One.

[19] Gnaiger E. (2020).

[20] Liu T., Chamkha I., Elmér E., Sjövall F., Ehinger J.K. (2025). Antibiotic-induced mitochondrial dysfunction: exploring tissue-specific effects on HEI-OC1 cells and peripheral blood mononuclear cells. BBA Gen Subj.

[21] Bol V., Bol A., Bouzin C. (2015). Reprogramming of tumor metabolism by targeting mitochondria improves tumor response to irradiation. Acta Oncol.

[22] Piel S., Ehinger J.K., Elmer E., Hansson M.J. (2015). Metformin induces lactate production in peripheral blood mononuclear cells and platelets through specific mitochondrial complex I inhibition. Acta Physiol (Oxford).

[23] del Portillo E.G., Olivares-Hernandez A., Gudino L.C. (2025). Evaluation of the effect of metformin as a radiosensitiser in solid tumours: a systematic review. Clin Transl Radiat Oncol.

[24] Dela R., Lemos Da Silva L., Beyer S. (2025). Not all tumors are alike: varying efficacy of FLASH across tumor types and oxygenation status in spheroid models. Br J Radiol.

