# Supplementary material for: Mitochondrial remodeling after conventional and ultra-high dose rate irradiation in head and neck carcinoma cells in vitro
Source: Clin Transl Radiat Oncol. 2026 Aug 6;61:101252. doi: 10.1016/j.ctro.2026.101252 (PMC13520611; doi:10.1016/j.ctro.2026.101252)
Supplement: Supplementary file 1 [file mmc1.docx]

**Supplementary Material (SM)**

**Figure S1: Additional aspects of effects on mitochondrial respiration with increasing doses of CONV** radiation**.** OXPHOS Complex I oxygen consumption (with ADP and Malate, Pyruvate and Glutamate present), LEAK oxygen consumption (after inhibition of the ATP-synthase with oligomycin), and ETS Complex II oxygen consumption (after inhibition of CI with Rotenone). The graphics and measurements were collected by DataLab software versions 6 and 7 (Oroboros Instruments, Innsbruck, Austria). MitoPedia nomenclature ^16^ . The bars represent mean values ± standard error of the mean (SEM); n=6 per condition and *p<0.05, **p<0.01, ***p<0.001 and **** p<0.0001 from FDR (most significative discovery).

**Figure S2: Additional aspects of effects on mitochondrial respiration with increasing doses of UDHR radiation.** OXPHOS Complex I oxygen consumption (with ADP and Malate, Pyruvate and Glutamate present), LEAK oxygen consumption (after oligomycin addition), and ETS Complex II oxygen consumption (after Rotenone addition). The graphics and measurements were collected by DataLab software versions 6 and 7 (Oroboros Instruments, Innsbruck, Austria). MitoPedia nomenclature ^16^ . The bars represent mean values ± standard error of the mean (SEM); n=6 per condition and *p<0.05, **p<0.01, ***p<0.001 and **** p<0.0001 from FDR (most significative discovery).

**Figure S3: Ratio OXPHOS/ETS from results obtained with CONV and UHDR with different total doses of radiation.** Results obtained after Succinate addition divided by the results obtained after FCCP maximum reaction. The graphics and measurements were collected by DataLab software versions 6 and 7 (Oroboros Instruments, Innsbruck, Austria). The bars represent mean values ± standard error of the mean (SEM); n=6 per condition and *p<0.05, **p<0.01, ***p<0.001 and **** p<00001 from FDR (most significative discovery).

**Figure S4: Gene Ontology of RNA-sequencing from HN4 cells after 48h of irradiation with 20Gy of total dose, with CONV and UHDR. a)** Biological Process description of the Downregulated and Upregulated Genes. **b)** Cellular Component description of the Downregulated and Upregulated Genes. **c)** Molecular Function description of the Downregulated and Upregulated Genes. The bar plot displays the top 10 most significantly enriched Gene Ontology (GO) terms associated with a set of genes. The y-axis of the bar plot shows these GO terms, while the x-axis represents the gene count (the number of genes that are associated with a specific GO term). The length of the bars indicates the gene count for each GO term. The color of the bars represents the adjusted p-value for each term.
